# Supplementary material for: The burden of disease and economic impact of sugar-sweetened beverages’ consumption in Argentina: A modeling study
Source: PLoS One. 2023 Feb 23;18(2):e0279978. doi: 10.1371/journal.pone.0279978 (PMC9949658; doi:10.1371/journal.pone.0279978)
Supplement: S1 File — (DOCX) [file pone.0279978.s001.docx]

Supplementary Material.

**Mathematical Model Structure Rationale**

To estimate the health and economic burden associated with the consumption of sugar-sweetened beverages in adults, the model used two main approaches: through a direct pathway, the direct effect of sugar-sweetened beverage consumption on cardiovascular disease and DBT2 was modelled; and through an indirect path the impact of the increase in BMI on diseases associated with overweight and obesity, secondary to the consumption of sugar-sweetened beverages, was modelled.

To model both pathways (direct and indirect) the model uses the population attributable fraction (PAF). The PAF is defined as the proportion of cases that can be attributed to a given exposure to a risk factor, which in this case corresponds to the consumption of sugar-sweetened beverages. For this purpose, our model uses a counterfactual scenario without exposure to the risk factor, i.e., without consumption of sugar-sweetened beverages. The equation used to calculate the FAP is as follows:

$$PAF = 1- \frac{1}{\sum_{i=1}^{n} RR(Xi;\theta k)}$$

Where $X_{i}$ represents the consumption of sugar-sweetened beverages (servings per day) by sex and simple age; 𝜃k corresponds to the relative risk value reported per one unit daily serving increase in sugar-sweetened beverage consumption for each simple age and sex, and finally, RR (Xi; 𝜃k) represents the specific relative risk for each disease by simple age and sex, according to consumption level.

Although both pathways rely on the PAF formula, they use a different approach to estimate the disease and economic burden attributable to beverage consumption.

**Direct pathway**

For this approach, we first estimated the PAF of incident, prevalent, and mortality cases for each of the diseases (cardiovascular disease and DBT2) using the following formula:

$$RR(Xi;\theta k) = exp- ln(\theta k \cdot Xi)$$

Once the values of FAP were obtained, they were multiplied by the total number of

incident, prevalent, and mortality cases of DBT2 and cardiovascular disease for the four countries for the year 2020. In this way, the number of incident, prevalent and mortality cases attributable to the consumption of sugar-sweetened beverages was obtained through this direct pathway for these diseases. Once the attributable burden of disease in terms of health was obtained, the economic burden attributable to the consumption of these beverages for DBT2 and cardiovascular disease was estimated using population and cost data for the year 2020.

**Indirect pathway**

To model the impact of sugar-sweetened beverage consumption on overweight, obesity, and diseases associated with these conditions in adults, the following formula was used to calculate RR which was then used to calculate FAP:

$$RR\left( X_{i};\theta_{k} \right)=\left\{ \begin{aligned} exp\left( \frac{ln\left( \theta_{k} \right)}{5}\cdot0.1\cdot X_{i} \right),BMI<25 \\ exp\left( \frac{ln\left( \theta_{k} \right)}{5}\cdot0.23\cdot X_{i} \right),BMI\geq25. \end{aligned} \right.$$

Where $X_{i}$ represents the consumption of sugar-sweetened beverages (servings per day) by sex and simple age; 𝜃k corresponds to the reported relative risk value per unit increase in daily serving in the consumption of sugar-sweetened beverages for each simple age and sex, and finally, RR (Xi; 𝜃k) represents the specific relative risk for each disease by simple age and sex, according to level of consumption.

***Table S1. Sources and Magnitude of Effect of SSBs on Diabetes Mellitus and Cardiovascular Disease, BMI, and of BMI on Chronic Disease Outcomes***

| **Risk factor** | **Type of effect estimate** | **Parameter** | **Source** |
| --- | --- | --- | --- |
| **SSB-BMI** | **Linear effect** | **Adults: - BMI<25, 0.10 (95%CI, 0.05–0.15) kg/m2**  **- BMI ≥25, 0.23 (95%CI, 0.14–0.32) kg/m2**  **Per additional serving per day**  **Children: - 0.57 kg/m2 for a 1.7 servings/day** | **Khatibzadeh et al (2012), de Ruyter et al (2012), Ebbeling et al (2012)** |
| **BMI-health events** | **Relative risk** | **Data that varies according to health events, and is specific age and specific gender in adults. It does not include the association for Cardiovascular events and Diabetes** | **Stanaway, J. D., et al (2018). Global, regional, and national comparative risk assessment of 84 behavioural, environmental and occupational, and metabolic risks or clusters of risks for 195 countries and territories, 1990–2017: a systematic analysis for the Global Burden of Disease Study 2017. The Lancet, 392(10159), 1923-1994.  Relative Risk data available at** [**https://ghdx.healthdata.org/gbd-2017**](https://ghdx.healthdata.org/gbd-2017) |
| **SSB-Incidence of type 2 Diabetes** | **Relative risk** | **1.37 (95%CI 1.15, 1.63) per serving/day of SSB** | **Imamura et al (2015) cited in the manuscript** |
| **SSB-Cardiovascular mortality** | **Relative risk** | **1.08 (95% CI, 1.04 to 1.13) per serving/day of SSB** | **Yin et al (2020) cited in the manuscript** |
| **SSB-Cardiovascular incidence** | **Relative risk** | **1.08 (95% CI, 1.02 to 1.14) per serving/day of SSB** | **Yin et al (2020)** |
