# Supplementary material for: The burden of disease and economic impact of sugar-sweetened beverages’ consumption in Argentina: A modeling study
Source: PLoS One. 2023 Feb 23;18(2):e0279978. doi: 10.1371/journal.pone.0279978 (PMC9949658; doi:10.1371/journal.pone.0279978)
Supplement: Attachment — Submitted filename: SSBS Argentina - Responses to reviewers.docx [file pone.0279978.s002.docx]

Reviewer #1: This paper is a modelled study of the health and economic impact of SSB consumption in Argentina. There has clearly been much work into the development and execution of the model and the graphs and tables are great. With significant revision (minor and some major) and refinement of the use of the English language throughout, this paper has the potential to provide a good addition to the literature.

It is difficult to provide all details to such a complex modelling exercise however, a robust scientific paper is one that other research teams can obtain the same results through the same data sources, software, stakeholders, literature, reports etc. It is always useful to keep this in mind when doing scientific writing. Some specific considerations:

Thank you for these comments. We agree on the importance of the transparency and reproducibility of findings in research. We have edited the manuscript and refined the English language use, following your suggestions.

• Abstract:

Methods: The two last sentences should be in past tense. Please revise.

Thank you. We have already corrected these typos.

Results: It might be useful to say in the first instance of the results that ‘our model estimated that because of SSB consumption…..’.

Thank you. We totally agree with this suggestion and we have already corrected it.

• Introduction:

First paragraph, first sentence: needs citing.

Thanks. It is done now.

First paragraph, last sentence: would be good to include which SDG regarded prevention and control of NCDs (i.e. SDG 3 or 3.4).

Thanks. It is done now.

Second paragraph, first sentence, change the tense. So….’Worldwide, eight million deaths were attributed to overweight or obesity in 2019…… The sentence reads poorly. Please revise. Eg: Worldwide, eight million deaths in 2019 were attributable to overweight or obesity – two of the most critical determinants of NCD morbidity and mortality.’

Thanks. We have edited the sentence after your suggestion.

Second paragraph, second sentence: what are the gender differences? This is too general. Either give values or delete. Similarly, what are some of the economic costs you speak of? Some stats would be useful for the reader.

Thanks. To avoid excess in the number of words, we decided to omit the statement related to gender differences in the introduction. Also, we are now providing some statistics related to economic costs.

Third paragraph, second sentence needs citing. There appears to be a lot of duplication in information in this paragraph. Suggest revising and citing correctly throughout.

Thanks. We have cited these statements correctly now.

Fourth paragraph: Results from studies done in the past should be written in past tense. Please revise.

Thanks. We have edited it.

Fourth, fifth and sixth paragraphs need revising. For instance, the authors begin paragraph seven by saying children and adolescents consume 40% more SSBs, confectionery and then go on to give stats for adult obesity. Would be much better to give stats at this point for child and adolescent obesity instead of talking about it in the fourth paragraph.

Thanks. We relocated ENNyS2 data

Seventh paragraph: You need to spell out PAHO in full the first time you use it and then use acronyms.

Thanks. Done

Also, how have PAHO prioritized the need to reduce SSB consumption? It’s a very general statement and doesn’t really tell the reader anything, as it currently is. Suggest further work.

Thanks. We added some strategies highlighted in the PAHO document

• Methods

The authors describe in this section the two causal pathways in the model (ie. BMI and SSB consumption). However, there is no mention of how (or what) determines change in SSB consumption or how it directly effects overweight and obesity independently of BMI and this is an important and relatively new concept to understand. Some (cited) biochemical/pathological background from the global literature on this concept would be useful in the introduction to highlight just how damaging SSBs are. It would help build the argument of why modeling is important. What determined the change in consumption? Literature? It is unclear what the difference is between the baseline and counterfactual and why that has been chosen for the model. Some explanation of this would make the results much clearer for the reader.

Thanks for your comments.

- We now added a paragraph in the introduction explaining the effects of consumption of SSB on BMI and SSB on diabetes II and CVD independent of BMI.
- The model estimates a hypothetical change in the consumption of SSB. The baseline is the current consumption of SSB in Argentina, and the counterfactual is the zero consumption. This statement is now explained better in the estimation of the PIF: “The PIF estimates the proportional reduction in population disease (incidence or prevalence) or mortality would occur if exposure to a risk factor (SSB current consumption) were reduced to a minimal theoretical risk exposure scenario (zero consumption).”

Figure 1 is a good figure. Describing the other diseases is useful for the reader.

Thanks. We appreciate your comment.

First paragraph, fifth sentence needs more detail on the conceptualisation of the model. Did the authors have the policy diaglogue? Attend the dialogue? Or is the policy dialogue a report? Who hosted the dialogue? What was the dialogue on? How did these ‘activities’ help shape the model’s development? In what way? Did you follow a protocol? Did you have aims and objectives? What were the needs of the decision-makers? What policies are the ones you speak of to control SSB consumption?

Thank you for your comment. We have now cited the report of the policy dialogue carried out in the framework of this project where the answers to these questions can be found. (available on: <https://www.iecs.org.ar/wp-content/uploads/Informe-Policy-dialogue-Azucar.pdf>)

For the PIFs, does it estimate the proportional reduction in incidence or prevalence of disease or mortality? The second sentence in this paragraph needs revising.

Thanks for your comment. We are explaining better now this statement:

“The PIF estimates the proportional reduction in population disease (incidence or prevalence) or mortality would occur if exposure to a risk factor (SSB current consumption) were reduced to a minimal theoretical risk exposure scenario (zero consumption).”

Throughout the methods section: your study was done in the past so you need to write it in the past tense (e.g., ….secondly, we estimated the effect of BMI on disease incidence…..Finally we applied….. The model calculated…

Thanks. We have corrected it.

Fourth paragraph: should read ‘the estimation of the impact of SSB consumption….. please revise.

Thanks. We have corrected it.

What other sensitivity analyses did you estimate? Is the STATA code available for your analyses?

Thanks. We did not carry out multiple sensitivity analyses. Now we are stating: “We used Monte Carlo simulations for the sensitivity analysis” (instead of reporting “sensitivity analyses”).

As mentioned in the manuscript, the sensitivity analysis was programmed in STATA and Visual Basic. Programming codes are available upon request by the reader.

The authors indicate results were generated by 1000 iterations using the distribution of SSB consumption and RRs for each country. Is the model estimating outputs for more than one country or is this an error?

Yes, this was an error. We have now corrected it. Thanks for your comment.

• Epidemiological data

Please provide details of the data sources. What were the surveys? What were the international sources? Where did the authors obtain SSB consumption data from? What is the ‘latest nation risk factor survey’ actually called?

Thanks. We have edited this paragraph according to your suggestions.

Did the authors do any ‘fiddling’ within DISMOD?

No. DISMOD was mainly used to calculate case fatality rate values from incidence and mortality data of the included diseases.

Could an independent person repeat this method and get the same results? How did you ‘identify’ the RR values? Why did you choose the diseases you did and not others?

The section could be written in a much clearer way. Suggest revising. For instance, the last sentence about the RRs from GBD (which needs to be spelt out in full the first time it’s used) should go before you begin talking about ‘identifying RRs’, the readers then know where the authors obtained the RRs from and makes it clear that is how they ‘identified’ them (if indeed it was where the values for T2DM and CVD were obtained from, it’s really not clear).

Thanks for this comment. We have edited this paragraph according to your suggestions.

• Direct medical costs

The authors speak of macro- and micro-costing approaches but only describe the micro-costing approach. What is the macro-costing approach? Where do these concepts originate? Are they backed by theory? Previous modeling studies? Please provide more detail.

Thank you for your comment. This project was carried out not only in Argentina but also in three other Latin American countries. For the Argentina case (purpose of this manuscript), we only used a micro-costing approach. We made a mistake when writing this paragraph, but now we only mention the micro-costing method.

Fifth sentence: please either name the previous cost estimation projects, clinical guidelines and local experts or provide a reference for each for the reader.

We appreciate your comment. In line with our response to the previous comment, we agree we can be more specific about what was done specifically for the case of Argentina. All micro-costings were carried out between a research economist and at least one local medical expert for each disease. Local medical experts suggested the annual resources that these patients usually need. This information was based on their daily experience and, in some cases, on local practice guidelines (diabetes and obesity). Therefore we have edited this paragraph and added the citations for these two local guidelines.

• Results

Overall, the authors need to describe their findings in much more detail. It is clear they have gone to the trouble to make detailed tables, but the text descriptions to highlight the significant findings for each would improve this section a lot. Compare and contrast. For instance, the authors indicate boys were more affected than girls. By how much? How much higher was the 5-11 age group’s burden compared to the lowest? How much more affected were adults 18-44 compared to older groups? 47 million. Is that USD?

Thank you for your contribution. We have already added this information.

Table 1

Please provide a definition for the reader of the different grades of obesity, either in the text or in the table or as a table footnote. Different countries have different thresholds for obesity and it is important to provide explicit detail for all tables and their content. Nowhere in the paper have the authors given any definition of the different BMI groups and what constitutes underweight, overweight or anything about the different ‘grades’ of obesity. Please revise for those readers not familiar with BMI ranges. Similarly, the authors may like to explain the reason for not having BMI means for children? Doing so will give this table a lot more meaning and relevance.

Thanks. We have now explained that the division by BMI values is the same as the World Health Organization. Regarding the mean point BMI in children, this input was not used by the model since the impact of SSB consumption through BMI on other diseases was not modeled in children.

Table 2.

Please check the rounding of values for incidence, prevalence and mortality. It may be preferable to have rates per 100,000 as whole numbers when above 100. Similarly, for very low numbers (e.g., 0.0) it may be better to have increased decimal points.

Please ensure units for numbers are included in tables. Overweight and obesity have <18 in the ‘diseases’ column, but what is that? Age? BMI? It is unknown without units.

Are the costs First Year (Acute Event) per capita? If so, please make clear.

Thanks. We have edited this table according to your suggestions.

Table 3.

Please check your units. Some dollars have no $ signs. Also, what do the two values for the direct medical cost, USD (million) represent? Is one Argentinian dollars and one USD? If so, please make this very clear for the reader within the table or as a footnote.

Or is it per capita/ for the whole population? As it is, no one knows.

Thanks. We now explain in the footer of the table that we first present the value attributed to the consumption of sugar-sweetened beverages for both health outcomes and direct costs. We also offer the percentage that this value represents of the total number of events or deaths or direct costs that this disease brings to Argentina (not only what is attributed to sugary drinks).

In this case, the total direct cost in the denominator represents all the expenditures in Argentina for this disease.

Be careful with the text. First sentence after Table 3 shows – 639,000 which makes it look like a negative number but it’s just punctuation. Could you use brackets instead or use the dash properly. Could you add up T2DM and CVD costs etc to show even more significant results and the significance of multiple morbidities?

Thanks. It was edited as you suggest.

Figures 2 & 3

These are nice graphs, particularly Figure 3. Where did you source the data from? This should be included in your paper.

Thanks. We appreciate your comment. Figure 2 and 3 show findings estimated through the model.

• Discussion

Overall, the discussion needs revising to improve the flow of information. There are some interesting points the authors make, but it’s adhoc and disjointed.

Paragraph 1, use past tense for studies already done. So, ‘In this study, we estimated….’

Thanks. It is done.

Paragraph 3: What is BASTA? Please spell out acronyms if only being used once or twice.

Thanks. It is the acronyms of a previous model published in the field in Argentina (B.A.S.T.A., for “Bebidas Azucaradas, Salud y Tarifas en Argentina”. We have now corrected it.

Paragraphs 4 & 5: Why are the authors discussing the strengths and limitations in the middle of your discussion? These go at the end of your discussion.

Thanks. It is done.

Paragraph 6: Why do countries in Latin America have a high consumption of unhealthy products?

Thanks. It is done.

Paragraphs 7 & 8: These would be better in the introduction.

We consider that what is related to the policies in this field in Argentina and the region can be better addressed in the discussion section. Also, the second reviewer suggested not addressing the issue of implemented or unimplemented policies in the introduction section.

The paper has some interesting, significant findings. With revision, it will be much improved and useful for fellow modelers. All the best with the revision.

Thanks. We appreciate your comments. We totally agree that your suggestions help a lot in improving this manuscript.

Reviewer #2: Thank you for the opportunity to review this paper, entitled ‘The Burden of Disease and Economic Impact of Sugar-Sweetened Beverages Consumption in Argentina: a Modeling Study’. The authors estimate the burden of disease and costs imparted by SSB consumption in Argentina. The authors have used a strong modelling approach to estimate this, and both the rationale and modelling techniques appear to be sound. However, much of the manuscript should be restructured to increase comprehension and readability. Further, the authors may wish to consider their interpretation of the model, as rather than estimating the exact burden of SSBs, it instead estimates the reduced burden if consumption were reduced to a predetermined level. Finally, should this paper be accepted for publication, I suggest further editing for English language and readability.

Thanks. We appreciate your comments. We have worked on your revisions and also in editing English language and readability.

Abstract

- As with much of the results and the discussion, please reframe to highlight that the model does not specifically estimate the cases of disease and deaths caused by SSB consumption (for example, of the 4,425 deaths mentioned, in the real world they are not only caused by SSB consumption but reducing SSB consumption may prevent them).

Thanks. We agree. We have now corrected it.

- Reformulating SSBs is not a good policy option to highlight in the abstract. I suggest discussing SSB taxation, marketing restrictions, or food and nutrition policies broadly.

Thanks. We agree. We have now corrected it.

Introduction

- The introduction is too long, and the structure makes it difficult to identify the issue being discussed. I suggest restructuring the introduction into three paragraphs:

1) Overweight, obesity and NCDs are a serious health concern globally.

2) An unhealthy diet, and consumption of SSBs is a key driver of overweight, obesity and NCDs. These are increasing worldwide.

3) In Argentina, consumption of SSBs is both high and increasing, and this is leading to an increased burden of disease.

4) Study rationale and aims

- Discussion of current policies to address unhealthy diets in Argentina should be left for the discussion.

Thanks. We agree. We have edited it following your suggestions.

Methods

- I feel that adding additional subheadings to the methods might clarify the approach for an audience with less economic modelling experience. For example, different sections for each of the three stages of estimating health and economic outcomes.

Thanks. We agree. We have edited it following your suggestions.

- Instead of a separate section on data sources, please describe each data source as its use is first mentioned in the text.

Thanks. We agree. We have edited it following your suggestions.

- Please define DALYs when first used

Thanks. We have now corrected it.

Results

- The main epidemiological and economic parameters included in the model should be presented in the methods, not the results (assuming that these are derived from pre-existing data sets).

Thanks. We have now corrected it following your suggestion.

- Please edit the results to use people-first language. For example, instead of “Overweight and obesity burden children and adolescents the most”, use “Children and adolescents face the greatest burden of overweight and obesity”.

Thanks. We have now edited it.

- Similarly, cases of obesity are not attributed to children/adolescents – children/adolescents are living with overweight and obesity.

Thanks. We have now edited it.

- In table 3, please present ‘events’ before ‘deaths’

Thanks. We have now edited it.

- It is difficult to say that SSBs cause 23% of all cases on diabetes, as so many factors contribute to disease. Instead, what the model estimates is that, if no SSBs were consumed, 23% of diabetes cases would be prevented. Please edit the results throughout to reflect this.

Thank you for your comment. We have now corrected it following your suggestion.

- I suggest not comparing the burden of disease across different NCDs as this implies that some are more important than others. Simply say how many cases of each would be avoided if SSB consumption was reduced to 0.

Thanks. We have now edited it following your suggestion.

- Please reference the results of the included sensitivity analyses

Thanks. We are now referring to sensitivity analysis findings.

Discussion

- As with the results, please be careful about directly attributing costs/deaths to SSB consumption – the model simply estimates that if SSB consumption were reduced, a certain number of costs and deaths would be prevented.

Thanks. We agree, and we have corrected it in this and other sections.

- Please mode the strengths and limitations to later in the discussion (just before the conclusion).

Thanks. We have now edited it following your suggestion.

- The authors state (in the limitations section) that their estimates are likely an underestimation, but it is not clear why this is the case – is this because oral health was not included? Please explain further.

Thanks. We have now edited it following your suggestion.

- Overall, the discussion would be improved by additional sections on drivers of SSB consumption and further consideration of policies. The authors discuss many policy options for addressing SSB consumption in Argentina, they may want to condense this a little and instead include more discussion on a) why SSB consumption is high (there is some discussion of advertising and prices, but there should be more on this) and b) how to implement best-practice policies (notably how to limit SSB industry influence).

Thanks. We have now added information concerning those points.
