# Supplementary material for: The burden of disease and economic impact of sugar-sweetened beverages’ consumption in Argentina: A modeling study
Source: PLoS One. 2023 Feb 23;18(2):e0279978. doi: 10.1371/journal.pone.0279978 (PMC9949658; doi:10.1371/journal.pone.0279978)
Supplement: Attachment — Submitted filename: SSB IECS - Plos One submitted.docx [file pone.0279978.s003.docx]

The consumption of sugar-sweetened beverages is a growing public health problem. In this study, the authors analyze SSB-associated burden in Argentina, one of the countries with the highest consumption of SSB in the world.

The authors have tried to carefully model the health burden and costs of SSB use. However, given the complexity of modeling itself, the article contains little information on the process of obtaining the results. I believe that the article needs a major revision of the methodological aspects before considering its publication in Plos One.

Here is a detail of suggested changes:

Major aspects

- reviewer #1 already mentioned the importance of providing accurate and explicit information about how the modeling process was conducted, and the lack of information in this regard. I do not believe you have addressed this concern. Although I understand that space constraints precludes authors from providing extensive and detailed data, these data are still needed, and you should consider adding an appendix that contains details about your assumptions.l. This appendix should at a minimum address/include:
  - the list of all outcomes evaluated and their attached RRs (used in the PIF equation)
  - a clarification of how the modeling pathway worked. For example:
    - How exactly does BMI affect diabetes and CVD outcomes? (the link between direct and indirect pathways). Is not clear to me looking at Figure 1.
    - How did you avoid duplicity of events? For example:
      - you applied a risk of mortality attached to diabetes, but then another risk for CVD outcomes. Did you exclude the population of diabetics for the latest? If not, wouldn´t there be a duplicity of CVD events among diabetics?
      - In figure 1, it seems you applied an effect of BMI on CVD (then reflects events through the direct pathway), but also a BMI effect directly on outcomes, which also includes CVD events. Did you differentiate between incident and established CVD?
    - Are age and gender differences in results based solely on differences on SSB consumption? How do you account for the inherent differential risk of events among different age and gender groups?
    - Cost analysis: although is not explicitly mentioned, it seems the analysis only covers one year of avoided events and related costs. Nevertheless, in table 2, you include a column describing costs since 2nd year/follow up. How were they modeled? Was there any assumption regarding economic burden in previous years?
  - population size assumptions, by age and gender. Source of information for population inputs (like census)
- The paper models a scenario of not only a zero SSB consumption (as if they were withdrawn from market) but also no caloric compensation at all (an aspect that should be discussed at some point in the article, and it's currently not). Numbers are therefore strikingly high regarding SSB burden. Nevertheless, the discussion barely highlights the utopic nature of the modeled scenario, and I think it should be talked through in the discussion and highlighted in limitations.

Minor changes:

Introduction:

- 3rd paragraph, first sentence: it needs a reference.
- 3rd paragraph. The sentence “People who are overweight or obese are at greater risk of developing severe health problems, such as type 2 diabetes, high blood pressure, asthma, sleep disorders, liver disease, low self-esteem, and social and emotional issues” needs a reference
- Last paragraph reads: “This manuscript presents the results for Argentina obtained through a collaborative, more comprehensive project….”. More than what?

Methods:

- For what period of time is the intervention being conducted? It seems to be 2020 (for what it says in Results) but this should be clearly stated in Methods (and in the abstract)
- You used Monte Carlo simulations to get uncertainty intervals. I wouldn't call this a sensitivity analysis since you don't compare your results under a different assumption but rather estimate the uncertainty around the result you already got. In the same way, I wouldn´t call having reported UI a strength (as you do in discussion), since uncertainty around the main results is expected to be reported in almost any study.

Conclusion

- The last sentence reads: “This study is the first to assess the health and financial burden of SSB consumption in the country, including children and a wide range of attributable conditions.”. Nevertheless, and as you mentioned previously, the BASTA study already evaluated the health burden of SSB consumption among adults in Argentina. I believe this sentence is misleading and should be rephrased.
