# Supplementary material for: The burden of disease and economic impact of sugar-sweetened beverages’ consumption in Argentina: A modeling study
Source: PLoS One. 2023 Feb 23;18(2):e0279978. doi: 10.1371/journal.pone.0279978 (PMC9949658; doi:10.1371/journal.pone.0279978)
Supplement: Attachment — Submitted filename: Response to reviewers SSBs ARGENTINA.docx [file pone.0279978.s004.docx]

Reviewer #1: The authors have done an excellent job of revising the manuscript in light of the suggestions from both reviewers. It flows well, it is clear and great to see the amended version. Congratulations.

A few further minor corrections are required for accuracy, consistency and transparency:

• It looks like there’s a few instances where writing in the present tense have slipped through and it should be past tense. Suggest doing a final check.

Thanks. We have corrected these typos.

• I question your definition of a PIF. It looks like the definition of a PAF.

See:

Barendregt & Veerman (2009) Categorical versus continuous risk factors and the calculation of potential impact fractions. J Epidemiol Community Health. 64:209-212. doi:10.1136/jech.2009.090274

Thanks for your comment. We analyzed both terms,1,2 and we agree that the best definition of our analysis is the PAF, so we modified it accordingly.

• Your sources of epidemiological parameters require references. What might be useful is including a technical appendix where all the data sources are provided in a table.

Thanks. We have already included all the epidemiological references in the manuscript.

• Where did you get the definition of SSBs from? It needs a reference.

Thanks. We defined SSBs as the following ones: sports and energy drinks, sugar-sweetened sodas, sugar-sweetened fruit juices, and sugar-sweetened and flavored waters. This decision was based on a previous paper that assessed the disease burden attributed to SSBs (Singh, G. M., Micha, R., Khatibzadeh, S., Shi, P., Lim, S., Andrews, K. G., ... & Global Burden of Diseases Nutrition and Chronic Diseases Expert Group (NutriCoDE). (2015). Global, regional, and national consumption of sugar-sweetened beverages, fruit juices, and milk: a systematic assessment of beverage intake in 187 countries. PloS one, 10(8), e0124845.)

• You need to reference the exchange rates published by the Central Bank of the country. You have included it at the bottom of Table 2, it will be consistent to include it also in your text.

Thanks. We used the exchange rate published by the Central Argentinian Bank. We have corrected it within the manuscript. Also, now we are referencing it correctly in the manuscript.

• In your text regarding economic values, clarify the values are US dollars (e.g., US$ 40 million, etc.) Otherwise, it is open to interpretation, even though you indicate in the tables it is USD. You could also just highlight at the beginning of your results all monetary values are in USD. Just make it clear.

Thanks. Now we are just using one symbol, the $ symbol referring to USD.

• Something has gone wrong with ‘Deaths’ in table 3. Looks like an overlap or duplication.

Thanks. We do not see that duplication in our document. Maybe it occurs when converting from word to pdf.

• Discussion. Last sentence, third paragraph

What is the age group in numbers of the ‘youngest group’? The current definition tells the reader nothing. The reader is looking for detail (e.g.,” …..’incidence occurred in the youngest group (12-18 years), particularly among men……”

Thanks. We have edited it according to your suggestions.

All the best for the final hurdle. It will be a great paper and will attract much interest from public health researchers and policy makers alike.

Many thanks. We really appreciate your suggestions.

Reviewer #3: The consumption of sugar-sweetened beverages is a growing public health problem. In this study, the authors analyze SSB-associated burden in Argentina, one of the countries with the highest consumption of SSB in the world.

The authors have tried to carefully model the health burden and costs of SSB use. However, given the complexity of modeling itself, the article contains little information on the process of obtaining the results. I believe that the article needs a major revision of the methodological aspects before considering its publication in Plos One.

Here is a detail of suggested changes:

Major aspects

- reviewer #1 already mentioned the importance of providing accurate and explicit information about how the modeling process was conducted, and the lack of information in this regard. I do not believe you have addressed this concern. Although I understand that space constraints preclude authors from providing extensive and detailed data, these data are still needed, and you should consider adding an appendix that contains details about your assumptions.l. This appendix should at a minimum address/include:

- the list of all outcomes evaluated and their attached RRs (used in the PIF equation)

Thanks. We are now including a statement and respective reference in the supplementary material with links to the specific GBD RRs used.

- a clarification of how the modeling pathway worked. For example:

- How exactly does BMI affect diabetes and CVD outcomes? (the link between direct and indirect pathways). Is not clear to me looking at Figure 1.

Thanks for your comment. To avoid double counting for cardiovascular events, we just applied one RR value, which is the RR of the association between SSBs consumption and cardiovascular events (the same for diabetes). Since the RR of the association between SSBs consumption and cardiovascular events is not adjusted for BMI, somehow, the indirect association (through BMI) between BMI and cardiovascular (the same for diabetes) is also being considered. For that reason, we show in figure 1 two different arrows regarding the direct and indirect (through BMI) association between SSBs and cardiovascular events (the same for diabetes). We are explaining it better in the manuscript, and we modified Figure 1 to avoid a misunderstanding.

- How did you avoid duplicity of events? For example:

- you applied a risk of mortality attached to diabetes, but then another risk for CVD outcomes. Did you exclude the population of diabetics for the latest? If not, wouldn´t there be a duplicity of CVD events among diabetics?

Thanks. Your comment is really interesting. No, we are not double counting cardiovascular events. On the one hand, we assumed a RR value of 1.37 for diabetes type 2 **incidence (assuming that it** consequently affects prevalent cases and also mortality in the same way) per SSB serving day.

On the other hand, we assumed a RR of 1.08 (95% CI, 1.04 to 1.13) and 1.08 (95% CI, 1.02 to 1.14) per serving per day for incidence and mortality of cardiovascular diseases. This value came from a meta-analysis in which the primary studies made adjustments for main potential confounders:

“All studies adjusted for age, smoking, and physical activity (8–11, 13, 14, 16, 21, 23, 25, 48, 49). Most studies controlled for other RR factors, including BMI (8, 9, 11, 13, 14, 21, 23, 48, 49) (N = 9), education (8, 13, 14, 16, 23, 49) (N = 7), alcohol consumption (8, 9, 11, 13, 14, 16, 21, 23, 25, 48) (N = 10), total energy intake (8–11, 13, 14, 21, 23, 48, 49) (N = 10), and dietary quality (8–11, 14, 16, 21, 25, 48, 49) (N = 10). SSBs and LCSBs were mutually adjusted in 4 studies.” (Yin et al: <https://www.ncbi.nlm.nih.gov/pmc/articles/PMC7850046/pdf/nmaa084.pdf>)

As far as we know, these studies adjusted for main lifestyle potential confounders (many of which are also associated with diabetes developing) but did not adjust for diabetes particularly. However, our model did not count cardiovascular events attributed to diabetes, so we are not double-counting cardiovascular events.

- In figure 1, it seems you applied an effect of BMI on CVD (then reflects events through the direct pathway), but also a BMI effect directly on outcomes, which also includes CVD events. Did you differentiate between incidents and established CVD?

Thank you for your comment. Yes, the model estimates incidents of CVD events attributed to BMI and how many established CVD events are also attributable to BMI.

- Are age and gender differences in results based solely on differences on SSB consumption? How do you account for the inherent differential risk of events among different age and gender groups?

Thank you for your comment. Most of the model inputs are differentiated by age and gender: BMI, SSB consumption, population, epidemiological data (incidence, prevalence, mortality), and the RR of BMI on the events (included in the supplementary material). Regarding the parameters of RR of SSB on BMI, CVD, or Diabetes, no information disaggregated by age and gender was found. We are adding this statement in the methods section now.

- Cost analysis: although is not explicitly mentioned, it seems the analysis only covers one year of avoided events and related costs. Nevertheless, in table 2, you include a column describing costs since 2nd year/follow up. How were they modeled? Was there any assumption regarding economic burden in previous years?

Thank you for your comment. As you have correctly mentioned, the model presents the diagnostic analysis of one year. The cost values that refer to consecutive years in Table 2 have been added to differentiate the costs in the incident and prevalent cases in those health conditions known to have essential differential costs between the first and consecutive years. So, we applied the healthcare costs only to incident cases and the consecutive year healthcare costs to the prevalent cases.

We added a explanation in the cost section:

“In turn, the costs were differentiated in the first year and subsequent years to distinguish and approximate the difference in costs of incident cases and prevalent cases.”

And we added a clarification in the model structure section:

“We used a comparative risk assessment framework to estimate the health and economic effects of sugar-sweetened beverage (SSB) consumption **in one year**.”

- population size assumptions, by age and gender. Source of information for population inputs (like census)

Thanks. We have included this information in Table 1 (we use the last National Argentinian demographic source, as referenced)

- The paper models a scenario of not only a zero SSB consumption (as if they were withdrawn from market) but also no caloric compensation at all (an aspect that should be discussed at some point in the article, and it's currently not). Numbers are therefore strikingly high regarding SSB burden. Nevertheless, the discussion barely highlights the utopic nature of the modeled scenario, and I think it should be talked through in the discussion and highlighted in limitations.

Thanks. We totally agree with you. Now we are including a statement in the discussion section.

Minor changes:

Introduction:

- 3rd paragraph, first sentence: it needs a reference.

Thanks. We have added a reference now.

- 3rd paragraph. The sentence “People who are overweight or obese are at greater risk of developing severe health problems, such as type 2 diabetes, high blood pressure, asthma, sleep disorders, liver disease, low self-esteem, and social and emotional issues” needs a reference

Thanks. We have added a reference now.

- Last paragraph reads: “This manuscript presents the results for Argentina obtained through a collaborative, more comprehensive project….”. More than what?

Thanks. We have edited this sentence now.

Methods:

- For what period of time is the intervention being conducted? It seems to be 2020 (for what it says in Results) but this should be clearly stated in Methods (and in the abstract)

Thanks. We are mentioning this statement now. However, we would like to emphasize that there is no intervention here, since we are just estimating the disease and economic burden attributed to SSBs consumption. To do that, we are comparing the context in Argentina in 2020 with a hypothetical scenario of no SSB consumption.

- You used Monte Carlo simulations to get uncertainty intervals. I wouldn't call this a sensitivity analysis since you don't compare your results under a different assumption but rather estimate the uncertainty around the result you already got. In the same way, I wouldn´t call having reported UI a strength (as you do in discussion), since uncertainty around the main results is expected to be reported in almost any study.

Thanks. We have edited these sentences now.

Conclusion

- The last sentence reads: “This study is the first to assess the health and financial burden of SSB consumption in the country, including children and a wide range of attributable conditions.”. Nevertheless, and as you mentioned previously, the BASTA study already evaluated the health burden of SSB consumption among adults in Argentina. I believe this sentence is misleading and should be rephrased.

Thanks. We have edited this sentence now.
