# Supplementary material for: The burden of disease and economic impact of sugar-sweetened beverages’ consumption in Argentina: A modeling study
Source: PLoS One. 2023 Feb 23;18(2):e0279978. doi: 10.1371/journal.pone.0279978 (PMC9949658; doi:10.1371/journal.pone.0279978)
Supplement: Attachment — Submitted filename: SSB Arg R3 responses to reviewers.docx [file pone.0279978.s005.docx]

A rebuttal letter that responds to each point raised by the academic editor and reviewer(s). You should upload this letter as a separate file labeled 'Response to Reviewers'.

A marked-up copy of your manuscript that highlights changes made to the original version. You should upload this as a separate file labeled 'Revised Manuscript with Track Changes'.

An unmarked version of your revised paper without tracked changes. You should upload this as a separate file labeled 'Manuscript'.

Deadline: Dec 02 2022 11:59P

Please use the space provided to explain your answers to the questions above. You may also include additional comments for the author, including concerns about dual publication, research ethics, or publication ethics. (Please upload your review as an attachment if it exceeds 20,000 characters)

Reviewer #1: Well done to the authors for improving this paper. There are only minor comments that require addressing:

Abstract

• If you have changed to describe a PAF, then you need to correct the wording in the abstract (it still says you’ve used the population impact factor).

**Response**: Thanks, we have edited it now.

Second page of introduction, fourth paragraph:

• The text indicates adults consume 85 litres of SSB per year. In the paragraph above that, it says children and adolescents consume 40% more SSBs that adults. Does that mean children and adolescents are consuming nearly 120litres per year?

**Response**: Yes. It can also be seen in Table 1.

Fourth page, first line:

• The authors need to correct the first line on this page to say…..’or mortality that would occur…….’

**Response**: Thanks, we have edited it now.

• Fourth line: the authors need to correctly say PAF, not population impact factor, and again, in the third paragraph on the same page.

**Response**: Thanks, we have edited it now.

Fifth page:

• Second paragraph: A reference needs to be included for the defining of SSBs

**Response**: Thanks, we have added it now.

• Second paragraph from bottom: is it possible to superscript the BMI, like so: kg/m2?

**Response**: Thanks, we have edited it now.

Sixth page:

• Second paragraph, third line: it should read ….’per SSB serving per day

**Response**: Thanks, we have edited it now.

• Direct medical costs: the authors have not included in the methods section if the costs were calculated from a societal perspective or a health perspective. Is this relevant for this micro-costing approach (not knowing what a micro-costing approach is).

**Response**: Thanks for this comment. We have clarified it now.

Table 3

• Although the authors have indicated there’s no problem with the ‘deaths’ row in Table 3, there still seems to be a doubling up of values over each other.

**Response**: Thanks. However, we do not see any overlap or duplication in Table 3 of our document. Maybe the Journal staff could help us with it.

Sensitivity analysis

• In the second round of reviewing, reviewer 3 mentioned uncertainty analysis using bootstrapping is not sensitivity analysis. It appears this has not been corrected?

**Response**: Thanks, we have edited it now.

Figures – great graphs!

A general comment: The paragraph's spacing is inconsistent throughout. Please check.

Also, the authors use the word ‘information’ to describe data. Would it be more academic to describe it as data? Finally, it may be useful to get a final proofread - there are still typos.

**Response**: Thanks, we appreciate those suggestions. We have edited it accordingly.

**Reviewer #3:** Thank you for the opportunity to review this revised version of the manuscript.

We appreciate the changes the authors have made with regards to our previous comments, and appreciate the inclusion of the supplementary material.

Nevertheless, we've noticed that the list of all outcomes evaluated and their attached RRs (used in the PAF equation) is still incomplete (for example: there is still no information regarding how outcomes such as asthma, musculoskeletal conditions or other conditions were modeled. If this is too detailed, even for the appendix, please amend the answers “Yes - all data are fully available without restriction” and “All relevant data are within the manuscript”, and specify how interested people can contact you to request the missing information.

**Response**: Many thanks. It is in our best interest that the methodology used in this analysis is well understood. Therefore, consequently to your suggestion, we have added detailed information in the supplementary appendix. Now all the formulas used can be seen, and you can understand better how we have modeled the indirect way (through BMI). Likewise, although the last PAF formula that we shared is mathematically correct and coincides with our analysis, we considered that it might be clearer for the reader to see it expressed in another way. That's why you'll see that we've also made this edit to the manuscript.

Regarding figure 1, if we are understanding it correctly, wouldn't something like this be more accurate, with CVD and other conditions as intermediate outcomes?

If we understood it incorrectly, please rearrange it so that it is clearer.

**Response:** Thanks for this comment. We are wondering if you tried to share with us some graphics since you mentioned, "wouldn't something like this be more accurate".

We will explain this more clearly so that it can be understood more adequately. Figure 1 is a general conceptual scheme of the model. It tries to summarize and simplifies what, in complex terms, the model estimates. The RR values of the direct effect between SSB consumption and incidence, prevalence, and mortality of Diabetes/CVD were Not reported as adjusted for BMI. For this reason, and to avoid double counting, we did not add RR values between BMI and incidence, prevalence and mortality of Diabetes/CVD. However, as the RR of the direct effect between SSB consumption and these two diseases was Not adjusted for BMI, our model somehow considers the effect between BMI and Diabetes/CVD. That is why Figure 1 schematically shows these two effect arrows reaching both Diabetes and CVD. Since the last figure was not fully understood, we are sending a new figure that, in our consideration, better represents what has been explained.

Finally, you say “Regarding the parameters of RR of SSB on BMI, CVD, or Diabetes, no

information disaggregated by age and gender was found”. We understand that RR may not be disaggregated by age and gender. We may have not been clear in our previous comment, but what we are wondering is: How did you model the risk of CVD and other diseases inherently associated with both age and gender, independent of the added risk of diabetes, BMI, or other variables under study? (For example, CVD risk is different for a 70-year-old man in comparison with a 35-year-old woman, even if both have no diabetes and a BMI below 25). Please clarify that in the methods.

**Response**: Thanks for this comment. This is correct. But our model does not need CVD risk information for every age and gender. Our model uses data on mortality and events by disease cause for all ages for the year 2020 in Argentina. Then, as the PAF formula explains, the model estimates the proportion of these deaths and events attributable to SSBs. Although the RR values of the consumption of SSB and CDV were the same for all genders and ages, the number of events and deaths from CDV was definitely different for the different ages and gender in Argentina in 2020. Also, the average consumption of SSB differed between those groups.

On a minor note, you are still referring to Monte Carlo simulations as “Sensitivity analysis” (results section).

**Response**: Thanks, we have edited it now.

I think the manuscript has improved since the previous version but still needs these relatively minor clarifications. We look forward to your answers to our comments.

**Response**: Many thanks. We really appreciate your suggestions.
